# Nucleotide polymorphism-based study utilizes human plasma liposomes to discover potential therapeutic targets for intervertebral disc disease

**DOI:** 10.3389/fendo.2024.1403523

**Published:** 2024-08-15

**Authors:** Ding-Qiang Chen, Zhi-Qiang Que, Wen-Bin Xu, Ke-Yi Xiao, Nai-Kun Sun, Hong-Yu Song, Jin-Yi Feng, Guang-Xun Lin, Gang Rui

**Affiliations:** ^1^ Department of Orthopedics, The First Affiliated Hospital of Xiamen University, School of Medicine, Xiamen University, Xiamen, Fujian, China; ^2^ The School of Clinical Medicine, Fujian Medical University, Fuzhou, China

**Keywords:** intervertebral disk degeneration, causality, liposome, metabolite, Mendelian randomization

## Abstract

**Background:**

While intervertebral disc degeneration (IVDD) is crucial in numerous spinally related illnesses and is common among the elderly, the complete understanding of its pathogenic mechanisms is still an area of ongoing study. In recent years, it has revealed that liposomes are crucial in the initiation and progression of IVDD. However, their intrinsic mediators and related mechanisms remain unclear. With the development of genomics, an increasing amount of data points to the contribution of genetics in the etiology of disease. Accordingly, this study explored the causality between liposomes and IVDD by Mendelian randomization (MR) analysis and deeply investigated the intermediary roles of undetected metabolites.

**Methods:**

According to MR analysis, 179 liposomes and 1400 metabolites were evaluated for their causal association with IVDD. Single nucleotide polymorphisms (SNPs) are strongly associated with the concentrations of liposomes and metabolites. Consequently, they were employed as instrumental variables (IVs) to deduce if they constituted risk elements or protective elements for IVDD. Furthermore, mediation analysis was conducted to pinpoint possible metabolic mediators that link liposomes to IVDD. The inverse variance weighting (IVW) was the main analytical technique. Various confidence tests in the causality estimates were performed, including consistency, heterogeneity, pleiotropy, and sensitivity analyses. Inverse MR analysis was also utilized to estimate potential reverse causality.

**Results:**

MR analysis identified 13 liposomes and 79 metabolites markedly relevant to IVDD. Moreover, the mediation analysis was carried out by choosing the liposome, specifically the triacylglycerol (48:2) levels, which were found to be most notably associated with an increased risk of IVDD. In all, three metabolite-associated mediators were identified (3-methylcytidine levels, inosine 5’-monophosphate (IMP) to phosphate ratio, and adenosine 5’-diphosphate (ADP) to glycine ratio).

**Conclusion:**

The analysis’s findings suggested possible causal connections between liposomes, metabolites, and IVDD, which could act as both forecast and prognosis clinical indicators, thereby aiding in the exploration of the pathogenesis behind IVDD.

## Introduction

Lipid metabolism represents a complicated process for maintaining the body’s regular physiological functions. Disorders of lipid metabolism are linked to various human disorders, including cardiovascular and bone diseases (1). IVDD represents a degenerative condition that is notably widespread. As the proteoglycan and water within the nucleus pulposus (NP) gradually decrease, the intervertebral discs (IVD) between the vertebrae may rupture. Subsequently, the herniation of the NP exerts pressure on the spinal nerves, leading to a significant deterioration in the patient’s quality of life (2). Furthermore, IVDD is a primary contributor to numerous spine-related conditions, including spinal stenosis and chronic sciatica (3). As the global population ages rapidly, the prevalence of IVDD continues to be high, posing a significant challenge to both public health and socioeconomic development. Nevertheless, existing non-surgical therapies and surgical procedures alike have been found ineffective in reversing the condition of IVDD (4).

Lipid metabolism performs a crucial function in maintaining IVD stability. When lipid metabolic homeostasis is disturbed, the IVD microenvironment is impacted. Disorders of lipid metabolism are strongly associated with the development of IVDD (5). A retrospective clinical study involving 302 hospitalized patients demonstrated that age, high-density lipoprotein cholesterol (HDL-C), and triglycerides (TGs) can influence degeneration levels of patients with symptomatic degenerative lumbar spine without underlying disease (6). Another case-control study with 396 patients and 394 controls showed that abnormal blood lipids may be linked to higher risk of lumbar disc herniation (7). The excess mechanic load on the lumbar spine caused by excess weight due to disorders of lipid metabolism is a logical pathogenic explanation for IVDD (8). However, the presumption of obesity being associated with pain and degenerative disease as a result of joint overload contradicts the fact that non-weight-bearing joints present with similar degenerative disease and related pain (9). An annulus fibrosus (AF) with structural deterioration is intimately linked to IVDD. Unusual mechanical stress is a major factor in annulus fibrosus cell (AFC) apoptosis, which harms the AF structurally and aggravates IVDD (10), and the majority of scholars agree that obesity is a mechanical risk factor for IVDD (11, 12). Nonetheless, the literature indicates that there has been disagreement on its function (13–15). It is not possible to attribute these observed disparities to the mechanical consequences of obesity alone. Obesity has not just mechanical impacts but also metabolic and inflammatory ones (16, 17). It is now acknowledged that adipose tissue is an active organ with a variety of metabolic and inflammatory characteristics. Adipokines, which function both locally and across the body’s circulation to reach distant regions and exert pro- or anti-inflammatory effects, are distinct and specialized proteins secreted by adipocytes (18). Obese adipose tissue produces systemic pro-inflammatory substances at a low level over an extended period of time, indicating that obesity-related chronic low-grade inflammation may play a significant role in IVDD. Thus, mechanistically, purely mechanical loading cannot explain the association between obesity and IVDD.

Other possible mechanisms beyond the impact of mechanical strain on the development of IVDD need further investigation. Plasma lipids are typically measured by HDL-C, low-density lipoprotein cholesterol, TGs, and total cholesterol (TC); however, the versatility and range of circulating lipids have been much more understood because to the development of contemporary high-performance lipidomic methods. Lipid classes are more finely delineated and may improve risk assessment for IVDD compared with standard lipids (19). Ultimately, an improved knowledge of the biology of lipid metabolism and its link to the pathogenesis of IVDD could also lead to novel therapeutic options for IVDD.

Presently, the most convincing explanation for the relationship between elevated body fat and IVDD is the internal secretory function of fatty tissue. Obesity increases the impaired cellular metabolism of free fatty acids, which promotes the development of IVDD (1). As a competitive inhibitor of arachidonic acid (AA), eicosapentaenoic acid (EPA) reduces inflammation by preventing the synthesis of lipoxygenase (LOX) and cyclooxygenase (CYC). In an alternative dietary control study, Napier et al. discovered that omega-3 fatty acids (n-3 FA) dietary supplementation lowers systemic inflammation and may even prevent the advancement of degenerative disc degeneration by lowering serum AA/EPA ratios. This was observed in a mice model of acupuncture-elicited IVDD (20). However, further exploration is needed to identify other possible metabolic factors linking liposomes to IVDD. The majority of existing research in this area has not conclusively established whether the liposomes examined have a direct causal connection to IVDD. Therefore, the search for IVDD-causing liposomes and the metabolite mediators that play an essential role between them could contribute to the mechanistic study of IVDD and it aids in making clinical judgments prior to the development of serious spinal conditions.

Luckily, the increasing attention on IVDD in the elderly population, genome-wide association study (GWAS) data related to IVDD has been concluded, uncovering genetic insights linked to the condition. Therefore, the possible causative relationship between liposomes, metabolites, and IVDD was assessed using MR techniques. A two-step MR approach was also used to identify possible metabolic mediators between liposomes and IVDD. As gametogenesis adheres to Mendel’s principles of inheritance, the distribution of parental genes to offspring occurs randomly in accordance with Mendelian genetics. Consequently, genetic types might not correlate with elements that affect the accuracy of observational research. MR techniques are capable of differentiating diseases from their underlying causes, thereby preventing the misinterpretation of causal relationships (21). Consequently, employing genetic variants associated with the target characteristic as IVs, MR emerges as a highly dependable analyzing technique that shows promise in determining exposure-outcome causality, eliminating confounding variables, measuring inaccuracies and correcting causal misinterpretations (22–24). Furthermore, genetic variation must be extracted from exposure- and outcome-related information in order to use two-sample MR designs, which enhances the statistical strength in confirming causal relationships between exposures and outcomes (25, 26).

## Materials and methods

### Study design

This research utilized the TwosampleMR software package. SNPs act as genetic proxies for inferring the influence of exposures on outcomes. Three presumptions should be met by genetic tools in MR analysis: SNPs are linked to the exposures, they are not connected with confounding factors that could interfere with the causality between the exposures and the outcomes, and their relationship with the outcomes are mediated exclusively through the exposures.

### Data sources

Summary statistics for liposomes (covering 4 major lipid categories: glycerolipids, glycerophospholipids, sphingolipids, and sterols) were obtained from a large-scale GWAS study by Linda Ottensmann and his team, which performed univariate and multivariate genome-wide analyses of 179 lipids from 7174 Finnish individuals. The GWAS catalog database provides access to the data for download (accession codes GCST90277238-GCST90277416) (19). Summary statistics for metabolites were obtained from a series of large GWASs study by Yiheng Chen and his team, which included 1091 metabolites and 309 metabolite ratios across 8299 participants. The GWAS catalog database provides access to the data for download (accession numbers from GCST90199621-90201020) (27). IVDD-related GWAS data from 184,683 subjects from European populations, including 20,001 IVDD cases and 164,682 controls, were sourced from the FinnGen repository, which contained a total of 16,380,337 SNPs.

### The process of selecting instrumental variables involved

SNPs were incorporated based on achieving a threshold of genome-wide significance (*P* < 1 × 10^-5^). For the inverse MR analysis, the IV significance threshold for IVDD was defined as 5×10^-8^. To avoid strong linkage disequilibrium (LD), an LD threshold of r^2^ < 0.001 was established. Additionally, the selection was limited to SNPs with an F-value greater than 10 to minimize the risk of weak instrumental bias. (**Supplementary Tables 1, 2**; **Table 1**).

**Table 1:** Characteristics of significant SNPs for intervertebral disk degeneration on liposomes.

SNP	pval.exposure	chr.exposure	pos.exposure	samplesize.exposure	beta.exposure	se.exposure	id.exposure	effect_allele.exposure	other_allele.exposure	eaf.exposure	exposure	R2	F
rs12308843	4.36E-08	12	23974404	184683	0.0689	0.0126	finn-b-M13_INTERVERTEB	C	G	0.3103	intervertebral disk degeneration	0.002031938	376.0243903
rs3010043	4.73E-09	1	183942175	184683	-0.0836	0.0143	finn-b-M13_INTERVERTEB	G	A	0.7913	intervertebral disk degeneration	0.002308374	427.2991764
rs3135840	9.27E-10	4	1796539	184683	-0.0816	0.0133	finn-b-M13_INTERVERTEB	T	A	0.2631	intervertebral disk degeneration	0.002581902	478.062514
rs4148946	3.00E-08	10	73770073	184683	0.0647	0.0117	finn-b-M13_INTERVERTEB	T	C	0.554	intervertebral disk degeneration	0.002068632	382.8289073
rs4473430	2.42E-08	2	69582895	184683	-0.0651	0.0117	finn-b-M13_INTERVERTEB	T	C	0.5523	intervertebral disk degeneration	0.002095821	387.8711576
rs62099230	3.27E-11	18	50721712	184683	0.0785	0.0118	finn-b-M13_INTERVERTEB	A	G	0.404	intervertebral disk degeneration	0.002967542	549.6798979
rs6470763	6.82E-09	8	130720646	184683	-0.092	0.0159	finn-b-M13_INTERVERTEB	C	G	0.1652	intervertebral disk degeneration	0.002334523	432.1508852

chr, chromosome; pos, position; beta, effect estimate; se, standard error of beta; eaf, effect allele frequency; R2, explained phenotypic variability; F, F statistic;

F = R2 (N − K − 1)/(K (1 − R2)); K, number of SNPs; N, sample size; R2, explained phenotypic variability.

### Data analysis

Utilizing the IVW method can provide a reliable assessment of causal relationships between exposures and outcomes, provided that each genetic variant meets the instrumental variable criteria (28). The Egger and weighted median methods can yield trustworthy causal inferences for a range of genetic variants, utilizing pooled data and operating under more lenient conditions. Even if up to 50 percent the data comes from genetic variants that are null IVs, the weighted median estimation continues to deliver coherent causality assessments (29). Additionally, MR-Egger regression and the MR-pleiotropy residual sum and outlier methods were used to detect and correct for pleiotropy. MR-Egger regression techniques aggregate causal effect estimates from multiple individual variables to assess and adjust for potential pleiotropy imbalances (30). Weighted linear regression of genetic outcome data to genetic exposure data was the approach that MR-Egger used. The causal estimation is represented by the slope of the linear regression, while the interception reflects the average impact of multiplicative effects from genetic variations (31). The heterogeneity among SNP estimates was evaluated using the statistical measure known as Cochran’s Q (32). A *P*-value greater than 0.05 suggests that neither horizontal pleiotropy nor heterogeneity is present. Ultimately, a leave-one-out approach was applied to the sequential removal of each SNP in order to verify that none of the SNPs had a significant effect on the outcome.

### Primary analysis

**Figure 1:**
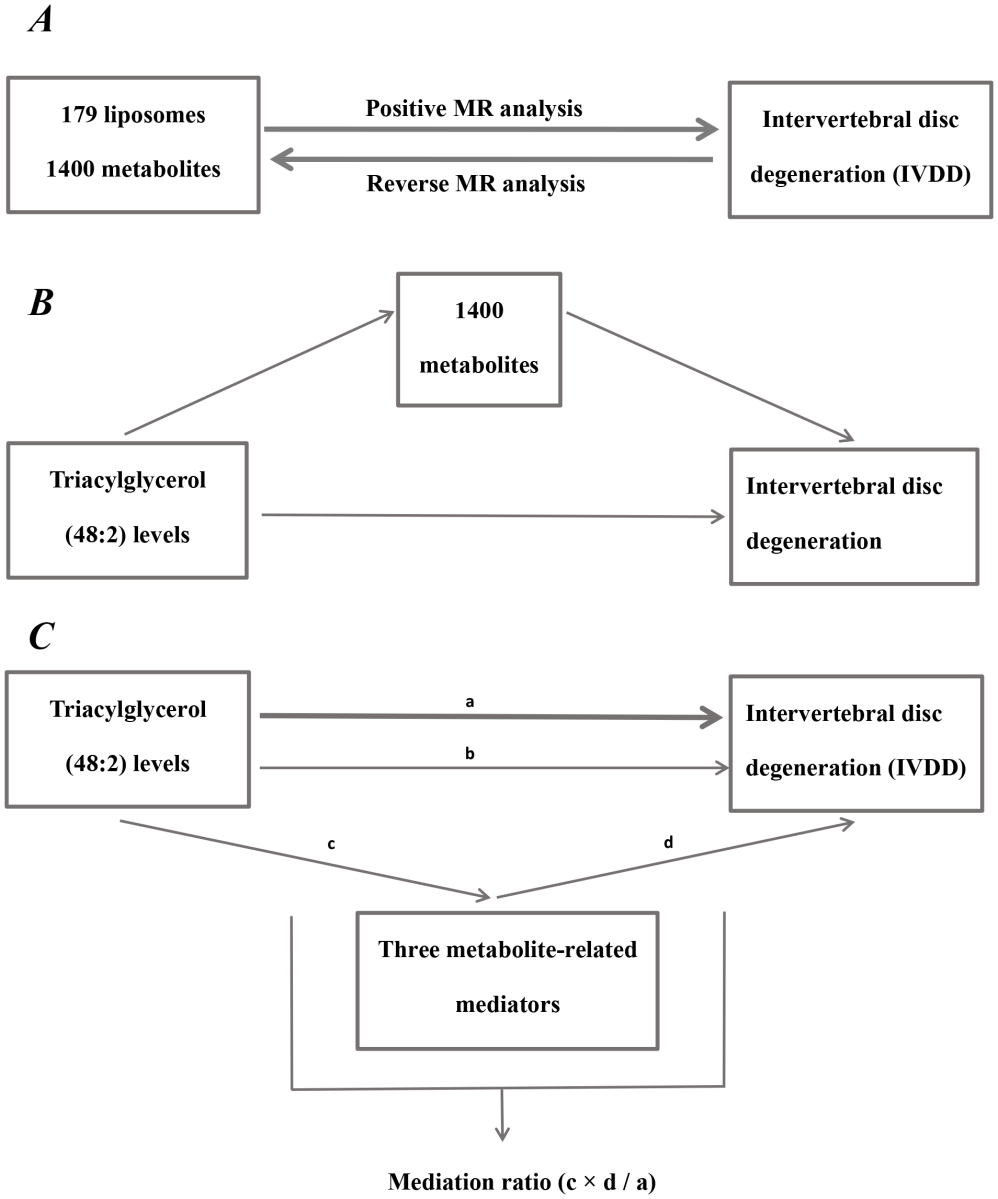
Diagram of analytical relationships. **(A)** Diagram of positive and reverse MR analysis between 179 liposomes and 1400 metabolites with IVDD. **(B)** Diagram of mediated MR analysis. Triacylglycerol (48:2) levels as the exposure factor, 1400 metabolites as mediators, and IVDD as the outcome. **(C)** Diagram of mediation ratio calculation. **‘**a**’** indicates the total influence of triacylglycerol (48:2) levels on IVDD. **‘**c**’** indicates the effect of triacylglycerol (48:2) levels on three metabolite-related mediators. **‘**d**’** indicates the effect of three metabolite-related mediators on IVDD. **‘**b**’** indicates direct effects = a - c × d (indirect impact). Mediation ratio = c × d**/**a.

First, to determine the causality between liposomes and IVDD or metabolites and IVDD (**Figure 1**), two-sample MR analyses were used. The primary analytical technique employed was the IVW technique. Additionally, validation was conducted using MR Egger, weighted median, simple mode, and weighted mode methods (33).

### Mediation analysis

Mediation analysis represents a novel technique for ascertaining if a variable mediates between two variables, which enables the construction of pathways from exposure factors to outcomes through mediators, aiding in the elucidation of the mechanisms through which exposure factors affect outcomes (34). For instance, both liposomes and metabolites have a significant causal impact on IVDD, with the liposome additionally having a significant causal effect on the metabolites. This setup establishes a triangular interaction where liposomes act as the exposure, metabolites serve as the mediator, and IVDD represents the outcome. The subsequent equation was applied: Mediation ratio = c × d/a. The total influence of liposomes on IVDD is dissected into two components: the immediate effect of liposomes on IVDD and the indirect influence mediated by the metabolites. The subsequent steps determine the percentage of mediating effect: initially, the impact of liposomes on metabolites is calculated to derive the value ‘c’; the next step involves computing the influence of metabolites on IVDD to ascertain the value ‘d’. The mediation impact percentage is subsequently determined by division of the indirect impact (c × d) by the total impact of liposomes on IVDD (a). (**Figure 1**). A larger percent of mediating impacts suggests that a greater portion of liposome impacts on IVDD are mediated through metabolites.

### Statistical analysis

This study was executed using R version 4.3.1. The “Forestploter” package was leveraged to create forest plots. Additionally, the “MRPRESSO” package was used for detecting outlier.

## Results

### MR analysis results of liposomes and IVDD

MR analysis showed a total of 13 liposomes were identified as significantly associated with IVDD, of which seven liposomes were risk factors, including diacylglycerol (18:1_18:3) levels, phosphatidylcholine (16:0_18:3) levels, and triacylglycerol (48:2) levels, etc. And six liposomes as protective factors, including phosphatidylcholine (14:0_16:0) levels, phosphatidylcholine (16:0_20:4) levels, sphingomyelin (d36:2) levels, etc (**Table 2**). Reverse MR analysis revealed a significant link between IVDD and one liposome phosphatidylcholine (O-16:0_20:3) levels (**Table 3**).

**Table 2:** Causal effects of liposomes on intervertebral disk degeneration.

Exposure	Outcome	Method	NSNPs	Pval	b	lo_ci	up_ci	OR	OR_lci95	OR_uci95
Sterol ester (27:1/22:6) levels	Intervertebral Disk Degeneration	MR Egger	23	0.449785537	-0.047918711	-0.169869035	0.074031613	0.953211269	0.843775314	1.076840847
		Weighted median	23	0.071462172	-0.068663623	-0.143325767	0.005998521	0.933640683	0.866471755	1.006016548
		Inverse variance weighted	23	0.024567029	-0.059510025	-0.111392674	-0.007627376	0.942226087	0.894587399	0.992401638
		Simple mode	23	0.788714782	0.020895484	-0.130081778	0.171872746	1.021115323	0.878023625	1.187526706
		Weighted mode	23	0.784909072	0.014280159	-0.087025546	0.115585865	1.014382608	0.916653678	1.122530897
Diacylglycerol (18:1_18:3) levels	Intervertebral Disk Degeneration	MR Egger	22	0.841075681	0.015365138	-0.132883402	0.163613678	1.015483789	0.875567176	1.177759233
		Weighted median	22	0.07857165	0.074216924	-0.008479107	0.156912954	1.077040416	0.991556739	1.169893775
		Inverse variance weighted	22	0.02743991	0.065194312	0.007249126	0.123139498	1.067366406	1.007275464	1.131042188
		Simple mode	22	0.185807059	0.110986349	-0.048041891	0.270014588	1.117379653	0.95309386	1.309983561
		Weighted mode	22	0.223858178	0.092987508	-0.052428617	0.238403633	1.097448026	0.948922055	1.26922139
Phosphatidylcholine (14:0_16:0) levels	Intervertebral Disk Degeneration	MR Egger	19	0.344911244	0.072019423	-0.073276873	0.217315718	1.074676217	0.929343484	1.242736395
		Weighted median	19	0.15065017	-0.063622919	-0.150387154	0.023141317	0.93835877	0.860374814	1.023411155
		Inverse variance weighted	19	0.0413156	-0.066602776	-0.130582739	-0.002622812	0.935566757	0.877583879	0.997380624
		Simple mode	19	0.434014701	-0.06381949	-0.220133006	0.092494026	0.938174334	0.802412065	1.096906588
		Weighted mode	19	0.469025493	-0.060081911	-0.219284219	0.099120396	0.941687396	0.803093431	1.104199233
Phosphatidylcholine (16:0_18:3) levels	Intervertebral Disk Degeneration	MR Egger	23	0.65448655	-0.034564452	-0.183785626	0.114656721	0.966026075	0.832114169	1.121488388
		Weighted median	23	0.102787931	0.074942969	-0.015090455	0.164976392	1.077822679	0.985022836	1.179365276
		Inverse variance weighted	23	0.044439415	0.064585868	0.001604179	0.127567557	1.066717171	1.001605466	1.136061614
		Simple mode	23	0.429240879	0.084285661	-0.120844348	0.28941567	1.087939631	0.886171883	1.335646802
		Weighted mode	23	0.325429004	0.095755788	-0.090836201	0.282347778	1.100490279	0.913167274	1.326239877
Phosphatidylcholine (16:0_20:4) levels	Intervertebral Disk Degeneration	MR Egger	24	0.13013339	-0.03852322	-0.086542936	0.009496497	0.962209362	0.917096172	1.009541732
		Weighted median	24	0.076560629	-0.033951334	-0.071525924	0.003623256	0.966618545	0.930972143	1.003629828
		Inverse variance weighted	24	0.045960097	-0.032517712	-0.064452779	-0.000582645	0.968005304	0.937580386	0.999417524
		Simple mode	24	0.42878099	-0.043796607	-0.150365709	0.062772495	0.957148615	0.860393265	1.064784568
		Weighted mode	24	0.098841262	-0.034922513	-0.074715321	0.004870294	0.965680241	0.928007633	1.004882173
Phosphatidylcholine (O-16:0_20:3) levels	Intervertebral Disk Degeneration	MR Egger	16	0.006126584	0.225248738	0.088292339	0.362205136	1.252634255	1.0923074	1.436493588
		Weighted median	16	0.071959018	0.078474876	-0.007005116	0.163954867	1.081636179	0.993019363	1.17816114
		Inverse variance weighted	16	0.00554003	0.085475558	0.025078208	0.145872908	1.089234938	1.025395312	1.157049127
		Simple mode	16	0.906793308	-0.008657073	-0.151150174	0.133836028	0.991380292	0.859718581	1.143205351
		Weighted mode	16	0.061309412	0.113119275	0.003505734	0.222732816	1.119765485	1.003511886	1.249486686
Sphingomyelin (d36:2) levels	Intervertebral Disk Degeneration	MR Egger	24	0.338868311	-0.060504388	-0.181801968	0.060793193	0.941289639	0.833766436	1.062679122
		Weighted median	24	0.140066187	-0.056237842	-0.130939867	0.018464184	0.945314274	0.877270526	1.018635701
		Inverse variance weighted	24	0.000287281	-0.096690669	-0.148948581	-0.044432756	0.907836785	0.861613417	0.95653992
		Simple mode	24	0.626967934	-0.032987251	-0.164240952	0.098266449	0.967550894	0.84853754	1.103256708
		Weighted mode	24	0.393496455	-0.042730452	-0.139039483	0.05357858	0.958169628	0.87019367	1.055039894
Sphingomyelin (d38:2) levels	Intervertebral Disk Degeneration	MR Egger	31	0.31898261	-0.051546411	-0.151185413	0.048092592	0.94975957	0.859688286	1.049267804
		Weighted median	31	0.421373415	-0.025968804	-0.089272628	0.037335019	0.974365485	0.914596195	1.038040726
		Inverse variance weighted	31	0.019611113	-0.055288302	-0.101723056	-0.008853548	0.946212314	0.903279675	0.99118553
		Simple mode	31	0.478609316	-0.046957	-0.175227897	0.081313897	0.954128424	0.839265733	1.084711331
		Weighted mode	31	0.631878214	-0.019380057	-0.097856153	0.059096038	0.980806529	0.906779332	1.060877121
Sphingomyelin (d42:2) levels	Intervertebral Disk Degeneration	MR Egger	38	0.848554686	-0.00869274	-0.097273015	0.079887535	0.991344933	0.907308264	1.083165243
		Weighted median	38	0.46398265	-0.023707876	-0.087161573	0.039745821	0.976570948	0.916528997	1.040546256
		Inverse variance weighted	38	0.041817716	-0.048205232	-0.09462634	-0.001784125	0.952938193	0.909712794	0.998217466
		Simple mode	38	0.526932209	0.050081762	-0.103598308	0.203761833	1.051357054	0.901587385	1.226006124
		Weighted mode	38	0.29159126	0.055920661	-0.046522323	0.158363646	1.057513779	0.954543252	1.171592162
Triacylglycerol (48:2) levels	Intervertebral Disk Degeneration	MR Egger	24	0.106048361	0.120252422	-0.019593905	0.26009875	1.127781493	0.980596808	1.297058165
		Weighted median	24	0.045882351	0.0847036	0.001547469	0.16785973	1.088394418	1.001548667	1.182770692
		Inverse variance weighted	24	0.006114398	0.079909912	0.022781075	0.137038749	1.083189481	1.023042546	1.146872587
		Simple mode	24	0.405868326	0.062287543	-0.081894357	0.206469443	1.064268323	0.92136929	1.229330168
		Weighted mode	24	0.259820507	0.07639007	-0.05320548	0.20598562	1.079383527	0.94818516	1.228735535
Triacylglycerol (49:1) levels	Intervertebral Disk Degeneration	MR Egger	25	0.609562849	0.035242725	-0.098167124	0.168652574	1.03587111	0.906497394	1.183708816
		Weighted median	25	0.022337169	0.089314087	0.012689403	0.165938772	1.093424033	1.012770255	1.18050082
		Inverse variance weighted	25	0.031312709	0.063876476	0.005728089	0.122024862	1.065960719	1.005744526	1.12978219
		Simple mode	25	0.161696862	0.120719681	-0.043150023	0.284589386	1.128308582	0.957767692	1.329216121
		Weighted mode	25	0.149027885	0.115098697	-0.036219891	0.266417284	1.121984168	0.964428201	1.305279617
Triacylglycerol (49:2) levels	Intervertebral Disk Degeneration	MR Egger	17	0.784613378	-0.020314298	-0.163404604	0.122776009	0.979890647	0.84924751	1.130631141
		Weighted median	17	0.122390038	0.068273563	-0.018348999	0.154896125	1.070658161	0.981818319	1.167536677
		Inverse variance weighted	17	0.032730104	0.06816972	0.005598775	0.130740664	1.070546986	1.005614478	1.139672185
		Simple mode	17	0.746137719	-0.027341979	-0.190040274	0.135356317	0.973028429	0.826925829	1.144944674
		Weighted mode	17	0.422051473	0.068284207	-0.09413872	0.230707134	1.070669557	0.910156496	1.259490323
Triacylglycerol (54:3) levels	Intervertebral Disk Degeneration	MR Egger	30	0.81516397	-0.014103229	-0.131240462	0.103034004	0.985995756	0.877006862	1.108529103
		Weighted median	30	0.237056075	0.042949496	-0.028246916	0.114145909	1.043885174	0.972148298	1.120915664
		Inverse variance weighted	30	0.006716964	0.06432523	0.017811802	0.110838659	1.066439181	1.017971378	1.117214639
		Simple mode	30	0.309226505	0.06141828	-0.054891589	0.177728149	1.063343596	0.946587763	1.194500551
		Weighted mode	30	0.296679268	0.048860784	-0.041254413	0.138975981	1.050074153	0.959584968	1.1490965

SNP, single nucleotide polymorphism; b, beta; OR, odds ratio; ci, confidence interval.

**Table 3:** Causal effects of intervertebral disk degeneration on liposomes.

Exposure	Outcome	Method	NSNPs	Pval	b	lo_ci	up_ci	OR	OR_lci95	OR_uci95
Intervertebral Disk Degeneration	Sterol ester (27:1/22:6) levels	MR Egger	7	0.75438629	0.355622052	-1.753045973	2.464290076	1.427068088	0.173245438	11.75513394
		Weighted median	7	0.56848866	0.076991805	-0.187622545	0.341606154	1.080033225	0.828927531	1.407205967
		Inverse variance weighted	7	0.76532834	0.035977287	-0.2002625	0.272217074	1.036632301	0.818515865	1.31287196
		Simple mode	7	0.319436811	0.222054462	-0.178945882	0.623054807	1.24863938	0.836151149	1.864615391
		Weighted mode	7	0.316598113	0.222054462	-0.176386877	0.620495801	1.24863938	0.838293604	1.85984993
Intervertebral Disk Degeneration	Diacylglycerol (18:1_18:3) levels	MR Egger	7	0.887155052	-0.119014374	-1.681468291	1.443439543	0.887795039	0.186100526	4.235238077
		Weighted median	7	0.815735958	-0.027831732	-0.261919814	0.20625635	0.972552002	0.76957273	1.229068235
		Inverse variance weighted	7	0.893042084	-0.01304125	-0.203147084	0.177064583	0.987043418	0.816158189	1.193708184
		Simple mode	7	0.692219217	-0.068799095	-0.393331046	0.255732856	0.933514209	0.674805319	1.29140769
		Weighted mode	7	0.693054774	-0.065876632	-0.377526972	0.245773709	0.93624636	0.68555471	1.278610202
Intervertebral Disk Degeneration	Phosphatidylcholine (14:0_16:0) levels	MR Egger	7	0.953785586	-0.053070149	-1.760609158	1.65446886	0.948313487	0.171940093	5.230301161
		Weighted median	7	0.889735629	-0.018726564	-0.283472664	0.246019536	0.981447689	0.75316371	1.278924559
		Inverse variance weighted	7	0.73125669	0.036398508	-0.171317826	0.244114841	1.037069044	0.842553745	1.276490916
		Simple mode	7	0.869726294	-0.035166857	-0.43787822	0.367544507	0.965444312	0.645404375	1.444184073
		Weighted mode	7	0.869027016	-0.035166857	-0.435703444	0.365369731	0.965444312	0.646809512	1.44104671
Intervertebral Disk Degeneration	Phosphatidylcholine (16:0_18:3) levels	MR Egger	7	0.953824162	-0.046426814	-1.541465417	1.44861179	0.954634424	0.214067174	4.257200524
		Weighted median	7	0.996580474	-0.000511994	-0.23466184	0.233637851	0.999488137	0.790838235	1.263186948
		Inverse variance weighted	7	0.593209475	0.049549041	-0.132251366	0.231349448	1.050797123	0.87612074	1.260299572
		Simple mode	7	0.973655695	-0.006133805	-0.35537456	0.343106949	0.993884968	0.700910861	1.409319479
		Weighted mode	7	0.950277199	-0.011403369	-0.355200793	0.332394054	0.988661403	0.701032667	1.39430217
Intervertebral Disk Degeneration	Phosphatidylcholine (16:0_20:4) levels	MR Egger	7	0.406708715	-0.687933907	-2.176974247	0.801106432	0.502613443	0.113384084	2.228004701
		Weighted median	7	0.941411815	0.008830679	-0.226668463	0.24432982	1.008869784	0.797185035	1.276765364
		Inverse variance weighted	7	0.745134751	-0.03004461	-0.211202605	0.151113385	0.970402243	0.809610019	1.163128532
		Simple mode	7	0.962231631	-0.009070613	-0.369221441	0.351080215	0.990970401	0.691272317	1.420601275
		Weighted mode	7	0.906443483	-0.020264974	-0.344296522	0.303766575	0.979938981	0.708718746	1.35495274
Intervertebral Disk Degeneration	Phosphatidylcholine (O-16:0_20:3) levels	MR Egger	7	0.153366002	1.35436911	-0.2237098	2.932448019	3.874315917	0.79954713	18.77353225
		Weighted median	7	0.748005822	0.045218361	-0.230649287	0.321086008	1.046256296	0.79401789	1.378624148
		Inverse variance weighted	7	0.042354549	0.215020491	0.007416502	0.42262448	1.239887304	1.007444073	1.52596116
		Simple mode	7	0.999294973	0.000179233	-0.381232534	0.381591	1.000179249	0.683019046	1.464612936
		Weighted mode	7	0.991765442	-0.002245968	-0.411445643	0.406953706	0.997756552	0.662691542	1.50223456
Intervertebral Disk Degeneration	Sphingomyelin (d36:2) levels	MR Egger	7	0.395389668	0.708347613	-0.78565092	2.202346147	2.030633094	0.455822901	9.046212364
		Weighted median	7	0.092434121	0.204651166	-0.03372611	0.443028442	1.227096938	0.966836276	1.55741663
		Inverse variance weighted	7	0.113990887	0.146529479	-0.035182999	0.328241956	1.157809061	0.965428728	1.388524894
		Simple mode	7	0.27913175	0.209714989	-0.135808459	0.555238437	1.233326498	0.873009833	1.742356378
		Weighted mode	7	0.251474938	0.209714989	-0.114217665	0.533647643	1.233326498	0.892063764	1.705140723
Intervertebral Disk Degeneration	Sphingomyelin (d38:2) levels	MR Egger	7	0.340895984	0.974630734	-0.840951431	2.790212898	2.650188405	0.431299976	16.28448637
		Weighted median	7	0.114701105	0.197206362	-0.047830978	0.442243703	1.217995363	0.953294901	1.556194943
		Inverse variance weighted	7	0.180869483	0.148357295	-0.068953862	0.365668452	1.159927258	0.933369742	1.441477245
		Simple mode	7	0.215833268	0.272555302	-0.113618823	0.658729428	1.313316086	0.892598129	1.932335602
		Weighted mode	7	0.184589165	0.257195459	-0.07914847	0.593539387	1.293297888	0.923902743	1.810384742
Intervertebral Disk Degeneration	Sphingomyelin (d42:2) levels	MR Egger	7	0.112912504	1.609408246	-0.033433791	3.252250284	4.999851672	0.967118941	25.84844084
		Weighted median	7	0.23322856	-0.165188771	-0.436788896	0.106411355	0.847733662	0.646107813	1.112279324
		Inverse variance weighted	7	0.860540251	0.021496652	-0.218325076	0.261318379	1.021729369	0.803864082	1.29864106
		Simple mode	7	0.364609067	-0.212175094	-0.636217471	0.211867284	0.808823065	0.5292907	1.235983839
		Weighted mode	7	0.327264034	-0.202838929	-0.575650913	0.169973056	0.816409731	0.562338716	1.185272915
Intervertebral Disk Degeneration	Triacylglycerol (48:2) levels	MR Egger	7	0.871152648	0.132207745	-1.385823392	1.650238883	1.141345404	0.25011777	5.208223835
		Weighted median	7	0.285134056	-0.138528874	-0.392554027	0.115496279	0.870638112	0.675329859	1.122430338
		Inverse variance weighted	7	0.246904614	-0.108194471	-0.29133728	0.074948338	0.897453051	0.747263598	1.077828467
		Simple mode	7	0.329468803	-0.213719568	-0.60848183	0.181042694	0.807574823	0.544176395	1.198466346
		Weighted mode	7	0.32222399	-0.210902956	-0.594164483	0.172358572	0.809852655	0.552023599	1.188103777
Intervertebral Disk Degeneration	Triacylglycerol (49:1) levels	MR Egger	7	0.876784406	-0.202256275	-2.631956172	2.227443622	0.816885554	0.071937602	9.276122472
		Weighted median	7	0.131633512	-0.229029429	-0.526767854	0.068708996	0.795305128	0.590510505	1.071124462
		Inverse variance weighted	7	0.11680968	-0.215895238	-0.485711588	0.053921113	0.805819717	0.61525923	1.055401341
		Simple mode	7	0.785095877	-0.067908711	-0.534638264	0.398820842	0.934345765	0.585881186	1.490066638
		Weighted mode	7	0.552594835	-0.143536007	-0.590880252	0.303808238	0.866289607	0.553839552	1.355009192
Intervertebral Disk Degeneration	Triacylglycerol (49:2) levels	MR Egger	7	0.584537602	-0.54906529	-2.391636515	1.293505934	0.577489343	0.091479853	3.64554522
		Weighted median	7	0.200247147	-0.177665526	-0.449535799	0.094204748	0.837222406	0.637924208	1.098784697
		Inverse variance weighted	7	0.494801408	-0.073128284	-0.283078613	0.136822046	0.929481585	0.753460554	1.146624083
		Simple mode	7	0.5874537	-0.124033975	-0.548296411	0.300228461	0.883349828	0.577933533	1.350167233
		Weighted mode	7	0.510179592	-0.135525716	-0.515031476	0.243980044	0.873256706	0.597481788	1.27631886
Intervertebral Disk Degeneration	Triacylglycerol (54:3) levels	MR Egger	7	0.466564903	0.753155579	-1.12093525	2.627246407	2.123690928	0.325974784	13.83561974
		Weighted median	7	0.8140446	-0.031118077	-0.290422732	0.228186578	0.969361107	0.74794732	1.256319706
		Inverse variance weighted	7	0.883245572	0.016505837	-0.203787579	0.236799253	1.016642811	0.815635611	1.267186708
		Simple mode	7	0.987453812	-0.002922126	-0.352340892	0.346496639	0.997082139	0.70304042	1.414104741
		Weighted mode	7	0.895409862	-0.024522732	-0.375012652	0.325967189	0.975775508	0.687280583	1.385369913

SNP, single nucleotide polymorphism; b, beta; OR, odds ratio; ci, confidence interval.

### MR analysis results of metabolite and IVDD

In the two-sample MR analysis, a total of 79 metabolites were found to be significantly linked to IVDD. Among these, 40 were identified as risk factors, such as N6-carbamoylthreonyladenosine levels, 2-hydroxy-3-methylvalerate levels, N-methyltaurine levels, 2-hydroxydecanoate levels, etc. Conversely, the remaining 39 metabolites were recognized as protective factors, including glucose to mannitol to sorbitol ratio, proline to trans-4-hydroxyproline ratio, mannose to mannitol to sorbitol ratio, etc (**Supplementary Table 3**).

### Findings from the mediation MR analysis

**Figure 2:**
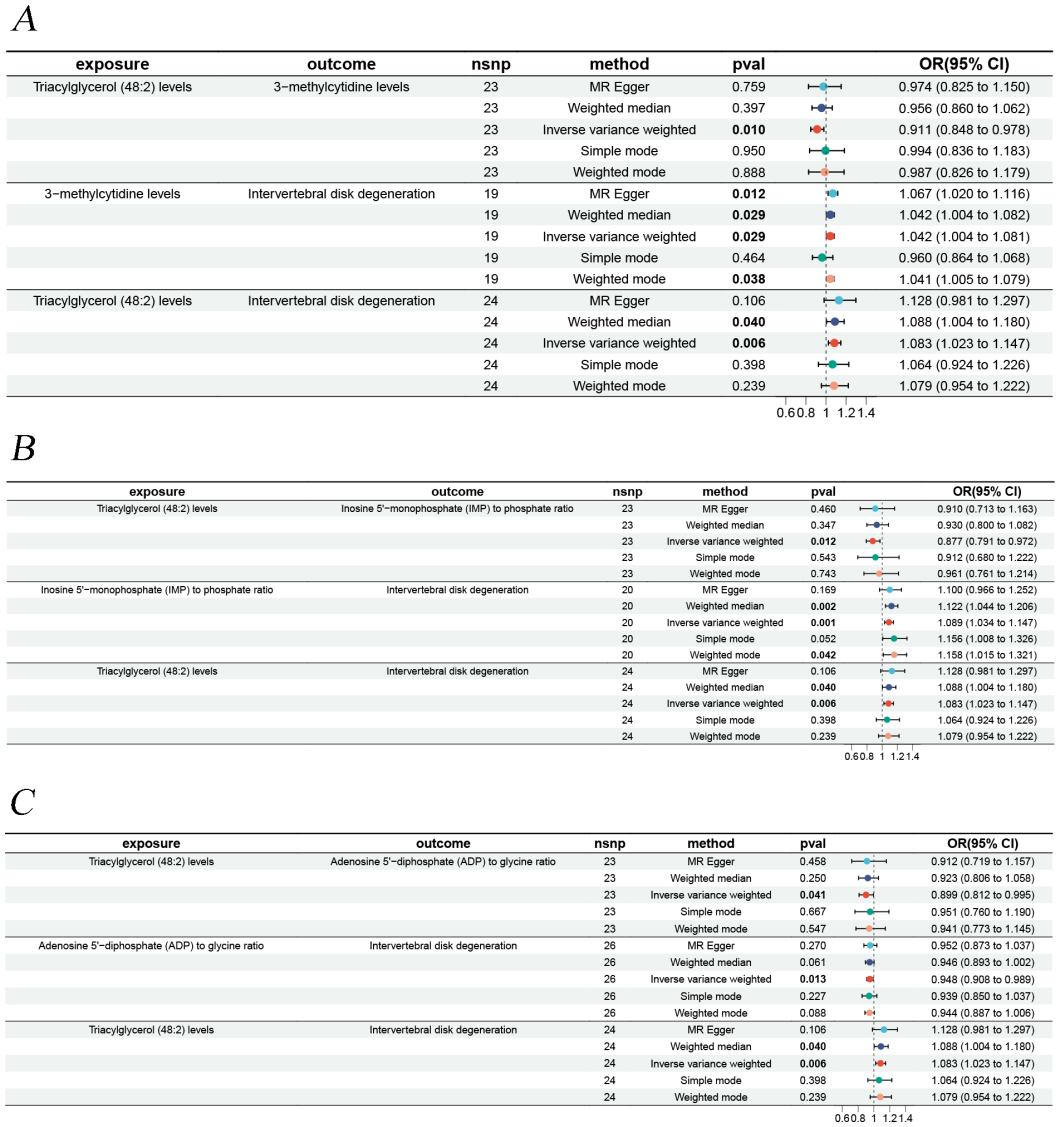
Forest plots illustrating the mediation analysis. **(A)** Triacylglycerol (48:2) levels impact on IVDD is mediated through 3-methylcytidine levels. **(B)** Triacylglycerol (48:2) levels impact on IVDD is mediated through inosine 5**’**-monophosphate (IMP) to phosphate ratio. **(C)** Triacylglycerol (48:2) levels impact on IVDD is mediated through adenosine 5**’**-diphosphate (ADP) to glycine ratio.

To explore how liposomes might be involved in the development of IVDD, we conducted a mediation analysis using MR, with metabolites serving as the mediating factors between liposomes and IVDD. Three distinct mediating associations were discovered. Triacylglycerol (48:2) levels influence IVDD risk via the three metabolites, with the mediation via the ADP to glycine ratio being consistent with the overall effect, with a mediating effect percentage of 7.13%. In contrast, the mediating effects of 3-methylcytidine levels and IMP to phosphate ratio were opposite to the overall impact (**Figure 2**).

### Sensitivity analysis

Sensitivity analyses revealed some MR findings were heterogeneous (**Tables 4**, **5**; **Supplementary Table 4**). The heterogeneity arises from the intrinsic nature of MR (35). When a gene is located on the same chromosome, it exhibits correlation without adhering to patterns of genetic independence in variation. Furthermore, IVs derived from various unit of analysis, studies, populations, etc., are probably displaying heterogeneity, which can impact the outcomes of MR analyses. The pleiotropy assessment disclosed instances of horizontal pleiotropy among certain liposomes and metabolites in relation to IVDD analysis (**Tables 4**, **5**; **Supplementary Table 4**). This suggests that segments of the IVs influence the outcomes through additional factors. However, the existing methodologies do not facilitate a comprehensive examination of the specific traits of every SNP. Consequently, we incorporated only those liposomes and metabolites that cleared the pleiotropy test in the following intermediary analysis (**Table 6**). Thus, the findings of this study maintain a high level of credibility. The leave-one-out test also indicated the reliability of the results (**Supplementary Figures S1****–****S3**).

**Table 4:** The result of heterogeneity and horizontal pleiotropic test of liposomes on intervertebral disk degeneration.

Exposure	Outcome	Heterogeneity test				Horizontal pleiotropic test		
		Method	Q	Q_df	Q_pval	egger_intercept	SE	p-value
Sterol ester (27:1/22:6) levels	Intervertebral disk degeneration	MR Egger	13.89077819	21	0.874244879	-0.001887375	0.009168416	0.838885853
		Inverse variance weighted	13.93315494	22	0.903834646			
Diacylglycerol (18:1_18:3) levels	Intervertebral disk degeneration	MR Egger	19.00932533	20	0.521219939	0.0074244	0.010373165	0.482430598
		Inverse variance weighted	19.52159682	21	0.551717965			
Phosphatidylcholine (14:0_16:0) levels	Intervertebral disk degeneration	MR Egger	16.44131439	17	0.492793193	-0.022163533	0.010804365	0.055969753
		Inverse variance weighted	20.64935046	18	0.297471342			
Phosphatidylcholine (16:0_18:3) levels	Intervertebral disk degeneration	MR Egger	23.70408013	21	0.307616485	0.013187297	0.00922477	0.167554406
		Inverse variance weighted	26.01085003	22	0.251216472			
Phosphatidylcholine (16:0_20:4) levels	Intervertebral disk degeneration	MR Egger	10.45134115	22	0.98173024	0.001790591	0.0054553	0.745841599
		Inverse variance weighted	10.55907582	23	0.987252964			
Phosphatidylcholine (O-16:0_20:3) levels	Intervertebral disk degeneration	MR Egger	6.128808613	14	0.963139607	-0.020330983	0.009122196	0.042731161
		Inverse variance weighted	11.09608043	15	0.745754117			
Sphingomyelin (d36:2) levels	Intervertebral disk degeneration	MR Egger	23.96836866	22	0.348889671	-0.006100061	0.009386935	0.52252004
		Inverse variance weighted	24.42845199	23	0.38041063			
Sphingomyelin (d38:2) levels	Intervertebral disk degeneration	MR Egger	38.53365869	29	0.110920823	-0.000703016	0.008410177	0.933955877
		Inverse variance weighted	38.54294328	30	0.136301808			
Sphingomyelin (d42:2) levels	Intervertebral disk degeneration	MR Egger	46.43768482	36	0.114116701	-0.006188593	0.00603024	0.311615738
		Inverse variance weighted	47.79625709	37	0.11006855			
Triacylglycerol (48:2) levels	Intervertebral disk degeneration	MR Egger	23.62189526	22	0.367356244	-0.00579535	0.009329717	0.540870407
		Inverse variance weighted	24.03619477	23	0.401806434			
Triacylglycerol (49:1) levels	Intervertebral disk degeneration	MR Egger	28.53886612	23	0.196087888	0.004648415	0.009905815	0.643300952
		Inverse variance weighted	28.81210226	24	0.227282638			
Triacylglycerol (49:2) levels	Intervertebral disk degeneration	MR Egger	13.1641753	15	0.589618702	0.013799066	0.010238945	0.197757538
		Inverse variance weighted	14.98048125	16	0.526068579			
Triacylglycerol (54:3) levels	Intervertebral disk degeneration	MR Egger	22.47005984	28	0.759078983	0.010511095	0.007351105	0.163819813
		Inverse variance weighted	24.5145764	29	0.703173206			

SE, standard error; df, degrees of freedom.

**Table 5:** The result of heterogeneity and horizontal pleiotropic test of intervertebral disk degeneration on liposomes.

Exposure	Outcome	Heterogeneity test				Horizontal pleiotropic test		
		Method	Q	Q_df	Q_pval	egger_intercept	SE	p-value
Intervertebral disk degeneration	Sterol ester (27:1/22:6) levels	MR Egger	9.950754905	5	0.07664267	-0.024211947	0.080886725	0.776730161
		Inverse variance weighted	10.12907117	6	0.119320733			
Intervertebral disk degeneration	Diacylglycerol (18:1_18:3) levels	MR Egger	1.219625282	5	0.942980785	0.008027362	0.059936251	0.898680519
		Inverse variance weighted	1.237562972	6	0.974988725			
Intervertebral disk degeneration	Phosphatidylcholine (14:0_16:0) levels	MR Egger	3.192639974	5	0.670314185	0.006777759	0.065507709	0.921615696
		Inverse variance weighted	3.203344991	6	0.782926181			
Intervertebral disk degeneration	Phosphatidylcholine (16:0_18:3) levels	MR Egger	3.777167475	5	0.581923327	0.007269991	0.057349975	0.904066159
		Inverse variance weighted	3.793236933	6	0.704633188			
Intervertebral disk degeneration	Phosphatidylcholine (16:0_20:4) levels	MR Egger	3.64001985	5	0.602314682	0.049832406	0.057117781	0.422873204
		Inverse variance weighted	4.401188796	6	0.62255437			
Intervertebral disk degeneration	Phosphatidylcholine (O-16:0_20:3) levels	MR Egger	5.18517898	5	0.393701438	-0.086297392	0.060531778	0.213293655
		Inverse variance weighted	7.292944164	6	0.294603443			
Intervertebral disk degeneration	Sphingomyelin (d36:2) levels	MR Egger	1.998047208	5	0.849415176	-0.042556438	0.057309584	0.491132851
		Inverse variance weighted	2.549458954	6	0.862891874			
Intervertebral disk degeneration	Sphingomyelin (d38:2) levels	MR Egger	7.374425976	5	0.194249446	-0.062591836	0.06964914	0.410013475
		Inverse variance weighted	8.565563991	6	0.199524505			
Intervertebral disk degeneration	Sphingomyelin (d42:2) levels	MR Egger	6.056462806	5	0.300763556	-0.120285626	0.06302179	0.114586655
		Inverse variance weighted	10.4690678	6	0.106238189			
Intervertebral disk degeneration	Triacylglycerol (48:2) levels	MR Egger	5.081601017	5	0.406002948	-0.018211279	0.058235846	0.767118836
		Inverse variance weighted	5.180988268	6	0.520818582			
Intervertebral disk degeneration	Triacylglycerol (49:1) levels	MR Egger	10.80620879	5	0.055360595	-0.001033773	0.093261663	0.991584589
		Inverse variance weighted	10.80647434	6	0.094544912			
Intervertebral disk degeneration	Triacylglycerol (49:2) levels	MR Egger	6.414942119	5	0.26791002	0.036051582	0.070681085	0.6317203
		Inverse variance weighted	6.748725717	6	0.344711329			
Intervertebral disk degeneration	Triacylglycerol (54:3) levels	MR Egger	7.857534253	5	0.164263946	-0.055798919	0.071888929	0.472740603
		Inverse variance weighted	8.80430279	6	0.184886754			

SE, standard error; df, degrees of freedom.

**Table 6:** The result of heterogeneity and horizontal pleiotropic test of 3 mediated relationships.

Exposure	Outcome	Heterogeneity test				Horizontal pleiotropic test		
		method	Q	Q_df	Q_pval	egger_intercept	se	pval
Triacylglycerol (48:2) levels	3-methylcytidine levels	MR Egger	20.78798429	21	0.471955826	-0.009663691	0.011039915	0.391290569
		Inverse variance weighted	21.55420628	22	0.486750455			
3-methylcytidine levels	Intervertebral disk degeneration	MR Egger	25.11094167	17	0.092253799	-0.011124871	0.00665853	0.11307325
		Inverse variance weighted	29.23426484	18	0.045590109			
Triacylglycerol (48:2) levels	Intervertebral disk degeneration	MR Egger	23.62189526	22	0.367356244	-0.00579535	0.009329717	0.540870407
		Inverse variance weighted	24.03619477	23	0.401806434			
Triacylglycerol (48:2) levels	Inosine 5'-monophosphate (IMP) to phosphate ratio	MR Egger	22.98822365	21	0.344606628	-0.005399542	0.016242271	0.742854387
		Inverse variance weighted	23.10920167	22	0.395585397			
Inosine 5'-monophosphate (IMP) to phosphate ratio	Intervertebral disk degeneration	MR Egger	8.276801264	18	0.974224776	-0.001598475	0.009806616	0.872334745
		Inverse variance weighted	8.303370154	19	0.983349345			
Triacylglycerol (48:2) levels	Intervertebral disk degeneration	MR Egger	23.62189526	22	0.367356244	-0.00579535	0.009329717	0.540870407
		Inverse variance weighted	24.03619477	23	0.401806434			
Triacylglycerol (48:2) levels	Adenosine 5'-diphosphate (ADP) to glycine ratio	MR Egger	16.4029829	21	0.74659883	-0.002064712	0.015775505	0.897115267
		Inverse variance weighted	16.42011271	22	0.794502667			
Adenosine 5'-diphosphate (ADP) to glycine ratio	Intervertebral disk degeneration	MR Egger	31.42489672	24	0.141905562	-0.000912331	0.008534701	0.915759523
		Inverse variance weighted	31.43985876	25	0.174864771			
Triacylglycerol (48:2) levels	Intervertebral disk degeneration	MR Egger	23.62189526	22	0.367356244	-0.00579535	0.009329717	0.540870407
		Inverse variance weighted	24.03619477	23	0.401806434			

SE, standard error; df, degrees of freedom.

## Discussion

This research explored the causality between liposomes, metabolites, and the risk of IVDD through MR analysis. We identified 13 liposomes (diacylglycerol (18:1_18:3) levels, phosphatidylcholine (14:0_16:0) levels, triacylglycerol (48:2) levels, etc.) and 79 metabolites (α-hydroxyisocaproic acid levels, butyrylpropionic acid levels, N-methyltaurine levels, etc.) might be linked to IVDD risk. Mediation analyses showed that triacylglycerol (48:2) levels could influence IVDD risk through three metabolites (3-methylcytidine levels, IMP to phosphate ratio, ADP to glycine ratio).

IVD is an avascular soft tissue structure situated in the intervertebral space. It is made up of the cartilaginous endplate, AF, and NP. IVDD, one of the most prevalent health issues worldwide, is considered to be a critical factor contributing to back and neck problems (36). Reduced levels of HDL and increased levels of TGs and LDL are the hallmarks of dyslipidemia. Lipid metabolism displays critical physiological functions in our body. Disorders of lipid metabolism can lead to obesity, hyperlipidemia, and hypercholesterolemia (37). A large-scale early cohort study involving 928 participants in the Wakayama Spine Study has revealed a possible link between disorders of lipid metabolism and IVDD (5). Zhang et al. found that clinically, age, BMI, and serum TGs were increased in the IVDD group by comparing IVDD grades with obesity-related elements in 128 volunteers and even once age and BMI are taken into account, TGs remain a significant risk factor for IVDD (38). Moreover, cell culture studies revealed that hypertriglyceridemia mediates disc cell apoptosis and matrix catabolism mainly through the MAPK signaling pathway, especially the ERK pathway (38). One of the risk factors for atherosclerosis is elevated blood TC. The four pairs of lumbar arteries and the central sacral artery are among the branch arteries of the abdominal aorta that may become blocked by atherosclerosis, which supplies blood to the lumbar spine. This obstruction further affects the blood supply to the IVD (39). Kauppila et al. found that advanced atherosclerotic manifestations of the aorta, particularly narrowing of the segmental arterial foramina above and below the IVDs, were associated with elevated IVDD grades, as assessed by routine autopsy of 86 lumbosacral spinal plain films and the corresponding abdominal aorta (39). The segmental arterioles’ stenosis was associated with elevated IVDD grades (40). High TGs play an essential function in IVDD development. Nevertheless, the underlying mechanisms still need to be completed. It is exciting that the present MR analysis identified three possible intermediary factors between TGs and IVDD.

IMP is a purine nucleotide essential to living organisms and enables animals to perceive fresh flavors (41–43). Zhang et al. found that oral administration of IMP to mice promoted the exogenous fatty acid uptake and conversion to TGs and enhanced the phosphorylation of liver IMP-activated protein kinase, resulting in adipose tissue hyperplasia (44). Obesity and being overweight are risk factors for lumbar radiculopathy and sciatica in both men and women, with a dose-response relationship, according to a meta-analysis that included data from 26 clinical investigations (45). Likewise, comparable findings were obtained by a recent meta-analysis that included ten cohort studies (46). This is consistent with our results. The results of the present mediated MR analysis suggest that TG levels cause a decrease in the IMP to phosphate ratio, which may be related to the negative feedback regulation caused by IMP facilitating the uptake and conversion of exogenous fatty acids to TGs.

ADP is an important signaling molecule that mediates various responses in cardiovascular, nervous, and other systems. Besides normal physiological processes, purinergic signaling facilitates numerous pathophysiological procedures, ranging from cell multiplication, polarization, cell motility, apoptosis, necrosis, vessel reshaping, and acute and chronic inflammation (47). Inflammation is strongly associated with the formation of thrombi, and platelets are vital mediators. ATP and ADP signaling have a crucial function in platelet activation. ADP triggers platelet aggregation via activation of P2Y1 and P2Y12 (48). Platelet activation and aggregation facilitate the attenuation of vascular rupture and bleeding in localized inflammatory areas, alleviating the ischemic and hypoxic conditions of tissues. Extracellular adenosine is also known as a “safety signal,” which inhibits the hypoxia-induced inflammatory response in ischemia and reperfusion (49). The conversion of extracellular ATP to adenosine is core to attenuating aseptic inflammation during ischemia/reperfusion injury. Experimental studies have shown that increasing the ATP catabolism to adenosine effectively attenuates tissue damage and aseptic inflammation in ischemia and reperfusion (50, 51).

Furthermore, some experimental studies have demonstrated the protective effects of adenosine signaling in ischemia and reperfusion models, e.g., adenosine or its analogs decrease the releasing of harmful oxidative metabolites from neutrophils after occupying specific receptors on neutrophils (52). Grenz et al. performed repetitive and nontraumatic occlusion of renal arteries in each adenosine receptor gene-targeted mouse. Then, Renal vascular A2B adenosine receptors protect the kidney against ischemia, as shown by measurements of certain parameters of renal function (53). The branches of the lumbar arteries, which begin at the base of the abdominal aorta, sustain the corpus lumborum. Nevertheless, arterial sclerosis usually occurs first in the lowest branches, leading to stenosis or occlusion of the lumbar artery segments (54, 55). As a result, some inflammation and damage caused by ischemia occurs. However, whether ADP can play a protective role against vertebral ischemic injury needs to be verified by more in-depth studies.

From a molecular biology perspective, the relationship between TG and IVDD can be explored at several levels. First, certain genes associated with TG metabolism may show aberrant expression in patients with IVDD, and such changes may affect disc cell metabolism and function by affecting TG synthesis or catabolism. For example, lipoprotein lipase and lipoprotein lipase-related protein play key roles in TG metabolism (56, 57), and their reduced expression in patients with IVDD may lead to TG accumulation, which affects intracellular lipid homeostasis and, consequently, disc health. Second, signaling pathways related to TG metabolism may be altered in IVDD, affecting cell proliferation, differentiation, and apoptosis. For example, the insulin signaling pathway plays an important role in regulating lipid metabolism and glucose metabolism, and its abnormalities may affect energy metabolism and anabolism of intervertebral disc cells, leading to degenerative changes (58). In addition, high TG levels may trigger cellular stress responses, such as endoplasmic reticulum stress, which affect disc cell function (59). Endoplasmic reticulum stress leads to the unfolded protein response, which activates the expression of a variety of stress-related genes and affects the balance of protein synthesis and degradation in cells, and prolonged endoplasmic reticulum stress may lead to cellular dysfunction and degeneration (60). TG accumulation may also trigger an inflammatory response, which may further exacerbate IVDD development. TG and its metabolites may affect the development of IVDD through the activation of inflammation-related signaling pathways, such as the NF-κB pathway, promoting the release of inflammatory factors, triggering an inflammatory response in the disc tissue, and further damaging the structure and function of the disc (61). Abnormal TG metabolism may also affect the synthesis and degradation of the disc’s extracellular matrix, leading to degenerative changes in the intervertebral disc. For example, matrix metalloproteinases (MMPs) play an important role in degenerative disc disease (62), and abnormal TG metabolism may affect the activity of MMPs, leading to degradation of the disc matrix and affecting disc stability and elasticity (63). Oxidative stress is also a key factor in the relationship between TG and IVDD. High TG levels may trigger oxidative stress (64), leading to damage and degenerative changes in intervertebral disc cells. Oxidative stress disrupts the intracellular antioxidant balance, leading to lipid peroxidation, protein oxidation, and DNA damage, which can further affect disc cell function and disc integrity (65, 66). Finally, genes associated with metabolic syndrome may play a role in IVDD, and these genes may affect both TG metabolism and disc health. For example, PPARγ (peroxisome proliferator-activated receptor γ) plays a key role in lipid metabolism and inflammatory responses, and aberrant expression of PPARγ may affect both TG metabolism and inflammatory responses in the intervertebral discs, which may contribute to the development of IVDD (67–69). By exploring the above molecular biological mechanisms, we can gain a deeper understanding of the relationship between TG and IVDD and provide a theoretical basis for future therapeutic strategies.

## Study strengths and limitations

This analysis has two strengths. First, it is the first study to investigate the relationship between liposomes and metabolites and the risk of IVDD using large-scale GWAS data, which eliminates confounders and reverse causality. This is highly beneficial in obtaining a thorough understanding of the relationship between liposomes and metabolites and the IVDD risk. Secondly, the bias was reduced through employing a two-sample study design, which involved using separate datasets for exposures and outcomes data, ensuring no intersection between the two sets of data. Nevertheless, there are certain aspects of this research that could be enhanced. Initially, the bulk of the data for this study was sourced from publicly accessible online databases; however, due to variations in platforms and geographic areas, some data might exhibit heterogeneity and may not entirely substitute for traditional research methods. Secondly, the IVDD data utilized in this study lacked specific details about the study subject, preventing a subgroup analysis based on various regions or gender. Consequently, the study outcomes might be limited in their applicability to populations from certain regions or specific genders. Finally, this study focused on analyzing causality from a genetic perspective, and the precise underlying mechanisms need to be explored in the future through necessary laboratory tests.

## Conclusion

Our comprehensive MR analysis identified 13 liposomes and 79 metabolites, which may have possible causal links to IVDD. Additionally, we discovered three intermediary associations between liposomes and IVDD. These metabolites and liposomes will be useful for IVDD mechanistic research as well as clinical diagnostics for prognosis and risk assessment.

## Data Availability

The original contributions presented in the study are included in the article/**Supplementary Material**. Further inquiries can be directed to the corresponding authors.
