# Supplementary material for: Nucleotide polymorphism-based study utilizes human plasma liposomes to discover potential therapeutic targets for intervertebral disc disease
Source: Front Endocrinol (Lausanne). 2024 Aug 15;15:1403523. doi: 10.3389/fendo.2024.1403523 (PMC11357925; doi:10.3389/fendo.2024.1403523)
Supplement: Supplementary file 2 [file Datasheet2.docx]

Fig. S1. Leave-one-out analysis of genetically predicted triacylglycerol (48:2) levels on intervertebral disk degeneration. The leave-one-out method is used to evaluate the excessive impact of a single SNP on MR analysis if the comprehensive effect of the remaining SNPs is consistent with the main effect after removing one SNP.

Fig. S2. Leave-one-out sensitivity analysis forest map. The leave-one-out method is used to evaluate the excessive impact of a single SNP on MR analysis if the comprehensive effect of the remaining SNPs is consistent with the main effect after removing one SNP. (A) Leave-one-out analysis of genetically predicted triacylglycerol (48:2) levels on 3-methylcytidine levels. (B) Leave-one-out analysis of genetically predicted triacylglycerol (48:2) levels on inosine 5'-monophosphate (IMP) to phosphate ratio. (C) Leave-one-out analysis of genetically predicted triacylglycerol (48:2) levels on adenosine 5'-diphosphate (ADP) to glycine ratio.

Fig. S3. Leave-one-out sensitivity analysis forest map. The leave-one-out method is used to evaluate the excessive impact of a single SNP on MR analysis if the comprehensive effect of the remaining SNPs is consistent with the main effect after removing one SNP. (A) Leave-one-out analysis of genetically predicted 3-methylcytidine levels on intervertebral disk degeneration. (B) Leave-one-out analysis of genetically predicted inosine 5'-monophosphate (IMP) to phosphate ratio on intervertebral disk degeneration. (C) Leave-one-out analysis of genetically predicted adenosine 5'-diphosphate (ADP) to glycine ratio on intervertebral disk degeneration.
