# Supplementary figures and images for: Nucleotide polymorphism-based study utilizes human plasma liposomes to discover potential therapeutic targets for intervertebral disc disease
Source: Front Endocrinol (Lausanne). 2024 Aug 15;15:1403523. doi: 10.3389/fendo.2024.1403523 (PMC11357925; doi:10.3389/fendo.2024.1403523)

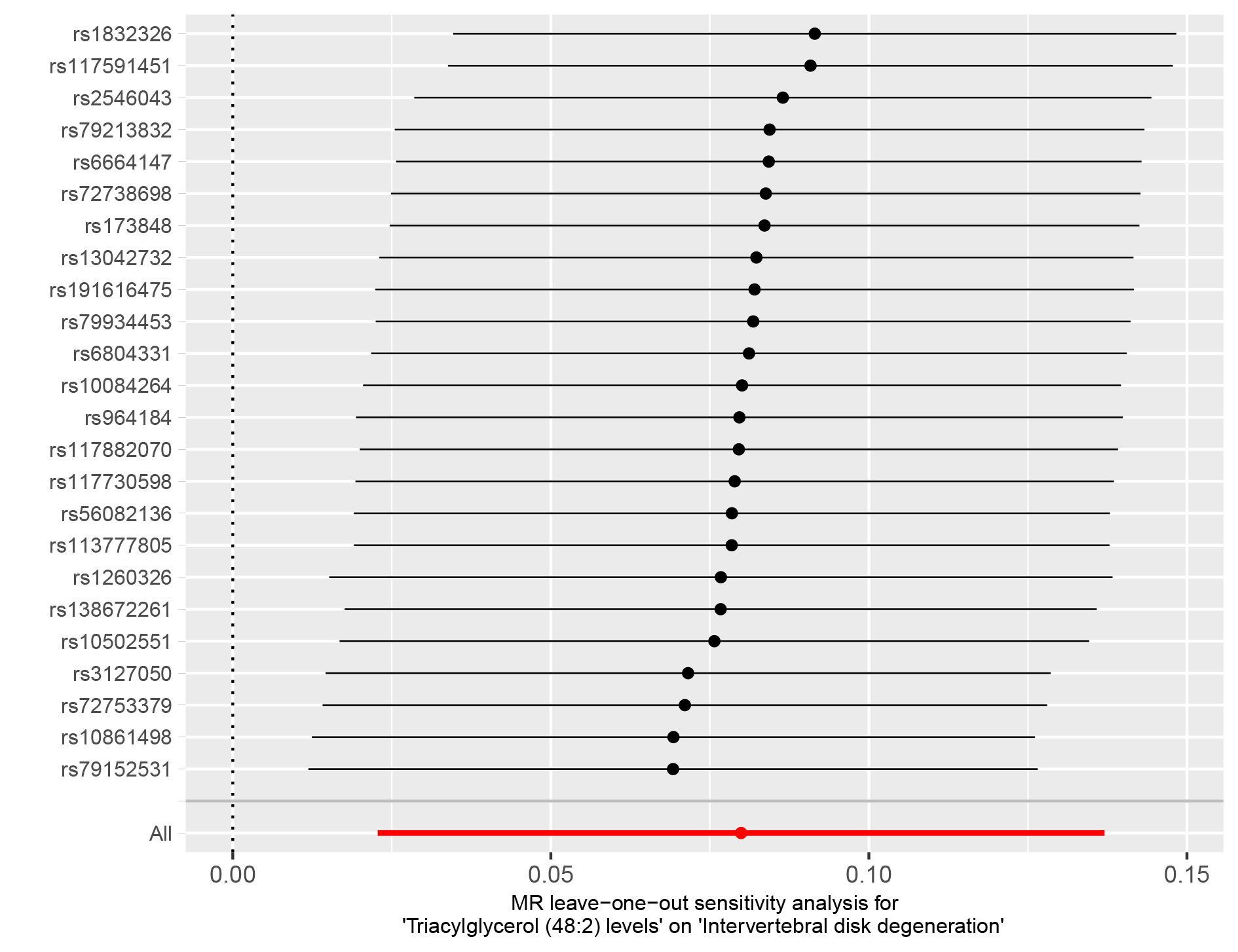

Supplement: Supplementary file 3 [file Image1.tif]

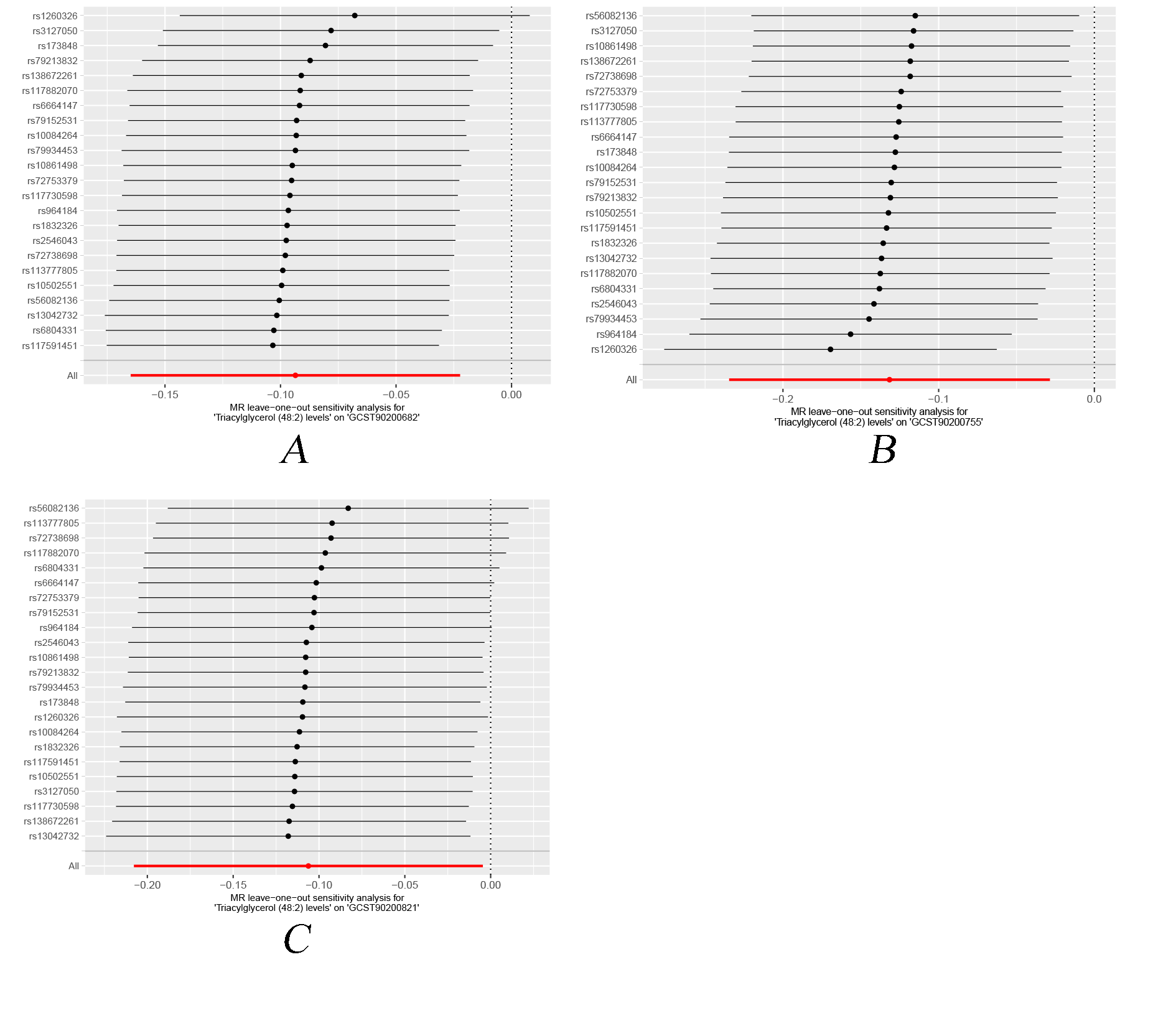

Supplement: Supplementary file 4 [file Image2.tif]

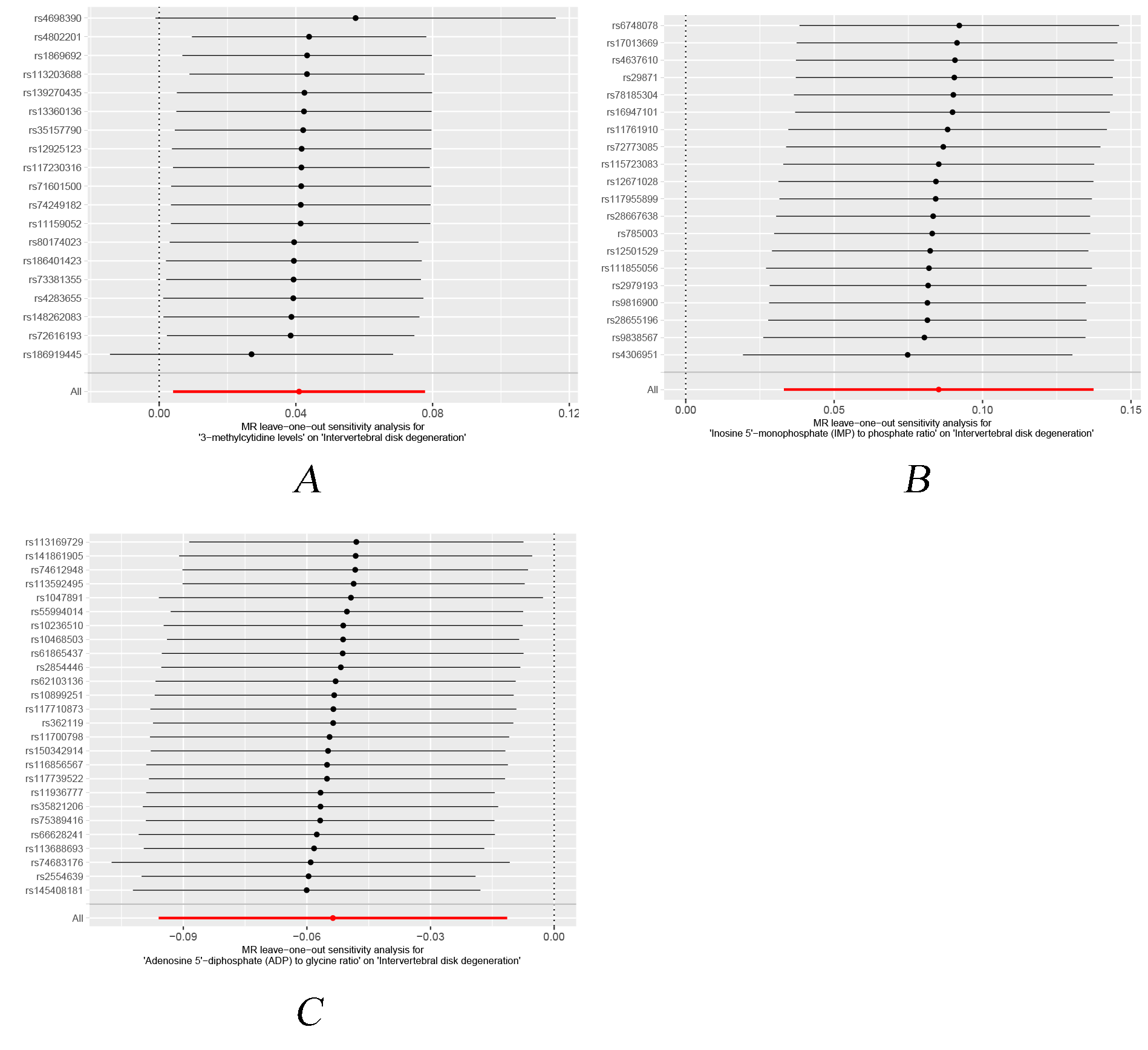

Supplement: Supplementary file 5 [file Image3.tif]
